# Challenges and Frugal Remedies for Lowering Facility Based Neonatal Mortality and Morbidity: A Comparative Study

**DOI:** 10.1155/2014/986716

**Published:** 2014-07-22

**Authors:** Hippolite O. Amadi, Akin O. Osibogun, Olateju Eyinade, Mohammed B. Kawuwa, Angela C. Uwakwem, Maryann U. Ibekwe, Peter Alabi, Chinyere Ezeaka, Dada G. Eleshin, Mike O. Ibadin

**Affiliations:** ^1^Department of Bioengineering, Imperial College London, South Kensington Campus, SW7 2AZ, UK; ^2^Departments of Community Health and Paediatrics, Lagos University Teaching Hospital, PMB 12003, Lagos 101001, Nigeria; ^3^Department of Paediatrics, University of Abuja Teaching Hospital, PMB 228, Abuja 900001, Nigeria; ^4^Department Obstetrics and Gynaecology, Federal Medical Centre Nguru, Nguru 630001, Nigeria; ^5^Federal Medical Centre Owerri, Owerri 460001, Nigeria; ^6^Department of Paediatrics, Federal Teaching Hospital Abakaliki, Abakaliki 480001, Nigeria; ^7^Federal Medical Centre Lokoja, Lokoja 260001, Nigeria; ^8^University of Benin Teaching Hospital, P.O. Box 1111, Benin-City 300001, Nigeria

## Abstract

Millennium development goal target on infant mortality (MDG4) by 2015 would not be realised in some low-resource countries. This was in part due to unsustainable high-tech ideas that have been poorly executed. Prudent but high impact techniques could have been synthesised in these countries. A collaborative outreach was initiated to devise frugal measures that could reduce neonatal deaths in Nigeria. Prevailing issues of concern that could militate against neonatal survival within care centres were identified and remedies were proffered. These included application of (i) recycled incubator technology (RIT) as a measure of providing affordable incubator sufficiency, (ii) facility-based research groups, (iii) elective training courses for clinicians/nurses, (iv) independent local artisans on spare parts production, (v) power-banking and apnoea-monitoring schemes, and (v) 1/2 yearly failure-preventive maintenance and auditing system. Through a retrospective data analyses 4 outreach centres and one “control” were assessed. Average neonatal mortality of centres reduced from 254/1000 to 114/1000 whilst control remained at 250/1000. There was higher relative influx of incubator-dependent-neonates at outreach centres. It was found that 43% of mortality occurred within 48 hours of presentation (d48) and up to 92% of d48 were of very-low birth parameters. The RIT and associated concerns remedies have demonstrated the vital signs of efficiency that would have guaranteed MDG4 neonatal component in Nigeria.

## 1. Introduction

The 25 years Millennium Development Goals target Number 4 (MDG4) of the United Nations required the reduction of the mortality rate of children below 5 years of age (U5MR) by a factor of 67% by the end of 2015. This translated to the saving of extra 520 babies per day out of the estimated 781 that died every day in Nigeria [1]. This was a huge and difficult task that required proactive thinking and development and implementation of revolutionary ideas given the antecedents of such a struggling low-resource country as Nigeria. All kinds of money-guzzling programmes that were based on foreign but internationally standardised methods have been executed in Nigeria from the inception of the MDG4 [2]. Various international donor agencies identified with a number of paediatrics healthcare needs donated money and collaborated with Nigerian Ministries of Health at Federal (FMoH) and State levels for the eradication of diseases and epidemics and general improvement of child health [2]. Large sums of money have been spent in a wide spread recruitment and engagement and reengagement of almost the same sets of expertise in a variety of changing patterns by FMoH. Unfortunately the U5MR has remained high as all these efforts have made little progress towards this ultimate goal.

The literature has revealed that a large number of babies die within the first 28 days of life (neonatal period) in many tropical countries. This includes Nigeria where over 660 newborn babies were estimated to die every day according to a more recently published report [3]. It has also been demonstrated that neonatal mortality rate (NNMR) accounted for up to 40% of the total U5 mortality [4]. This was quite significant than to be ignored as was realised by some Latin American countries [5]. By simple mathematics, it could be explained that any measures taken to reduce NNMR would contribute significantly towards the realisation of MDG4. However, most of the international and national collaborative programmes repeatedly targeted older children without significant impact on the neonates [5]. Unfortunately, even if the rest of older U5 children were all saved without a significant reduction in NNMR, the MDG4 target of 67% would still not be realised. Many years on from inception, Nigeria could not demonstrate significant MDG4 progress going by the ten-year timeline between Ibe's “increasing admission delivery” and Ogunlesi et al.'s “worsening hypothermia risks” [6, 7]. Kangaroo-mother-care (KMC) techniques of keeping babies warm may scarcely work for big neonates [8]. However extreme preterm babies (<29 weeks GA) or extreme low-birth-weight babies (<1000 g) might likely die without proper incubator intervention.

A typical Nigerian special-care-baby-unit (SCBU) could have a daily census of up to 45 neonates on admission at the same time of which 15 or more would be incubator-dependent very-preterm or extreme-preterm babies [7, 10]. Such common scenario implied a basic requirement of minimum 20 functional incubators at any given time for a standard Nigerian SCBU. Sadly no referral centre could demonstrate the availability of up to 4 functional incubators for a consecutive time period of two years [11]. It was rather common to see a large number of dysfunctional and obsolete incubators littering the hospital walkways, workshops, dump sites, and scrap yards whilst the SCBUs remained empty [12]. The unaffordability of reliable incubator systems in low-income settings was a well-known fact as a modern incubator sold in excess of £25,000 [13]. The maintenance experts of these mostly foreign products were unavailable. Poor spare parts supply chain and unstable and erratic power supply constituted some of the harsh operating conditions for the nondurability of these systems whenever any few could be procured. Since such large number of functional incubators must be available on a continuous long term bases to guarantee improved and sustainable neonatal survivability, Nigeria should have reconsidered other options in order to save its babies.

Individual referral centres in Nigeria could have adopted nonconventional measures to create new standards that might save more babies and hence enable the realisation of MDG4 target. The aim of this work was to investigate how remedies to some identified issues of concern might have changed practice and overall neonatal survival rate in few hospitals in Nigeria.

## 2. Materials and Methods

A collaborative medical outreach was launched in 2003 as a private research initiative to apply some willing Nigerian tertiary/referral hospital neonatal centres to develop affordable incubators and techniques that could tackle lack of functional systems at the centres [11]. The application of recycled incubator technology (RIT) developed gradually and became popular among a number of leading teaching hospitals scattered across the landscape of Nigeria [14]. In a resolution, the Committee of Chief Executives of Federal Tertiary Health Institutions (CCEFTHI) of Nigeria endorsed the new application and moved into collaboration in using this to save more babies in a few prospective Special Care Baby Centres in Nigeria [15]. The new application, though in insufficient number in these centres, provided continuous services that soon revealed the other practice deficiencies that contributed to poor outcome other than lack of incubators. Nine different parameters and situations of  “concern” were identified. These were individually experimented upon by the provision of prospective solutions before adoption as standard “remedies.” The concern remedies formed the various aspects of practice procedures in the collaborative outreach. All participating centres readily adopted the proposed concern remedies, albeit with varying degrees of implementation with which the participating hospitals distinguished themselves based on their various outcomes.

In order to investigate how these remedies might have corroborated with the level of practice outcomes from each centre, scores were assigned to various degrees of implementation of the concerns. A centre could score a maximum of 5 points on each of the concerns remedies if fully implemented or nothing if completely ignored. The various concerns are described as follows.

### 2.1. Concern-1: RIT Applied

The recruitment of hospital centres into this scholarly project followed a natural desire of the hospital management of each prospective centre to reduce their prevailing high neonatal mortality rate (NNMR). It was often difficult for them as standard incubators were unaffordable. Hence they assembled all their available old and obsolete incubator casings for recycling. Earlier publications demonstrated the trial, introduction, and standardisation of an incubator recycling technique that has safely been applied with high reliability and safety records for up to ten years [10–12]. The systems were often fully restored at costs that were smaller than 25% of the costs of modern incubators [11]. The recycled-incubator-technology (RIT) systems were constructed using internet-sourced generic components arranged by design and applied to the old casings [11]. The new low-cost systems so-produced were capable of 10 years of life expectancy and easily maintainable by locals [10, 12].

**Figure 1:**
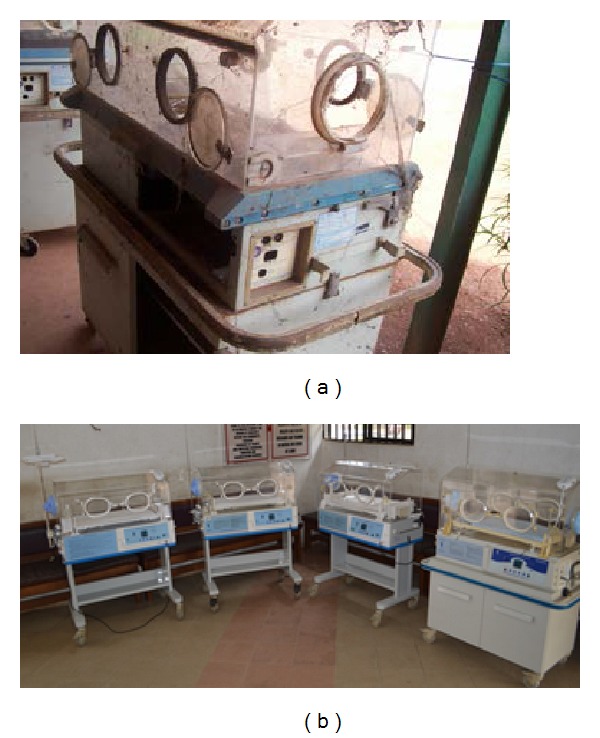
Incubators. (a) Casing recovered from a hospital dump site for recycling. (b) A fleet of  recycled systems just before commissioning at FMC Lokoja.

RIT incubators were built within the premises of the collaborating hospital using provided casings. Finished products have been applied in the main referral hospitals that presently serve up to 20 states in Nigeria (Figure 1).

The RIT was applied to extensively improve the capacity of outreach centres in terms of the number of functional incubators available to handle the high volume of admission delivery that was on the increase. A good example was the Lagos University Teaching Hospital (LUTH) that grew from no available functional incubator in January 2007 to become Nigeria's largest centre with 38 units of functional incubators and 8 units of neonatal Resuscitaires (total 46 units) by December 2013. These systems were distributed within 3 large hospital wards that looked after babies born within the hospital (inborn), babies referred from outside (outborn), and paediatrics surgical ward, respectively.

The Concern of incubator availability in each centre was scored as follows: 0–4 functional incubators received zero (0 pt), 5–9 (1 pt), 10–14 (2 pts), 15–19 (3 pts), 20–24 (4 pts), and 25 or more (5 pts).

### 2.2. Concern-2: Training

The introduction of RIT systems and the associated outreach amongst Nigerian hospitals particularly popularised attainment of sustainable and consistent presence of functional incubators in any Nigerian centre. Prior to this, the use of the very old and crude technique of “hot-water-bottles” was the most popular method of providing warmth to the neonate at many premier teaching hospitals in Nigeria. Many hospitals still practice this at the present in Nigeria.

**Figure 2:**
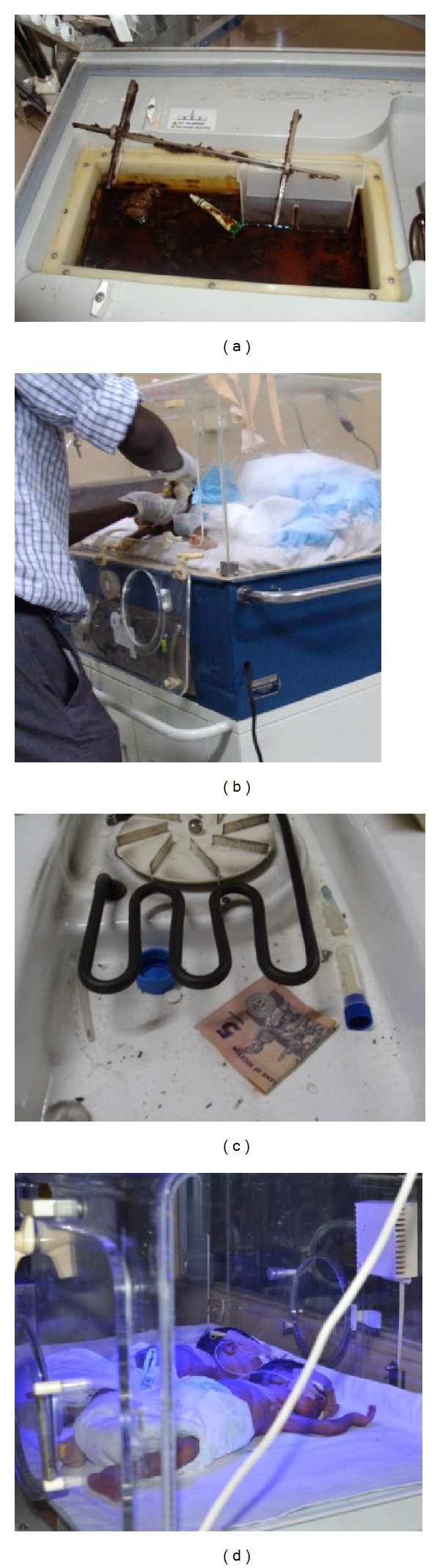
Few observable incubator bad practices. (a) A filthy humidifier chamber that had never ever been drained of water or cleaned. (b) Clinician using an operating incubator as a work bench for a very long procedure. (c) All sorts of materials left and concealed within a functioning incubator. (d) Multiple neonates sharing the same incubator.

The sustained presence of functional incubators at the centres soon exposed the other deficiencies of nursing and clinical skills associated with incubator care. It became necessary to develop two levels of elective courses for clinicians and nurses in order to instruct on the various unacceptable practices being exposed. Most of these awful practices ultimately led to infections and cross-infections, as primarily witnessed and evidenced from patient's case-notes, thereby impoverishing overall outcome (Figure 2). At the time of preparing this paper, over 1600 candidates had passed through level-1 course and up to 200 others had progressed to the level-2. This equipped candidates with a more advanced skill of patient-specific thermoneutral control in cases of very prematurity and very low birth-weight. A perceived improvement on overall outcome through these courses in some centres made the training become an important component for career progress for the staff of the centres. Courses were executed as frequently as the hospitals called for this and up to two streams in a year. Centres were given 1 pt for each course organised between April 2011 and March 2013 up to a maximum of 5 pts.

### 2.3. Concern-3: Capacity Expansion

**Figure 3:**
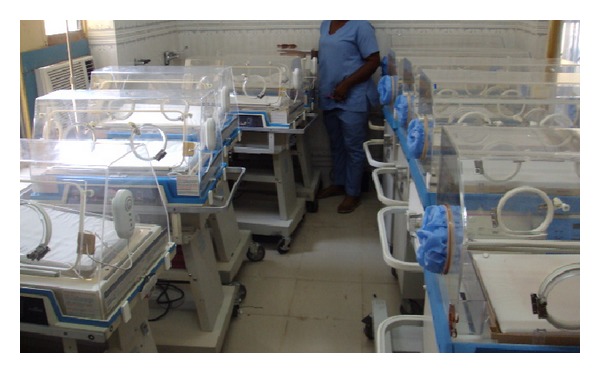
New RIT-fleet at UBTH Benin-City. Casings were sent from the USA through BRONDEK partnership Atlanta Georgia.

Many centres accepted RIT as affordable option of restoring proper neonatal incubation in the SCBUs. This led to the desire to increase their incubator capacities beyond what the available old/abandoned systems could number to adequately accommodate the increasing admission of incubator-dependent neonates. Hospitals were assisted to obtain casings of “used” or obsolete models at give-away prices through some foreign agencies. The obtained systems were then restored to functionality using RIT components and methods, hence making more incubators available for the babies (Figure 3). No assessment score was assigned to this as this application reflected in the scoring of Concern-1.

### 2.4. Concern-4: Research

**Figure 4:**
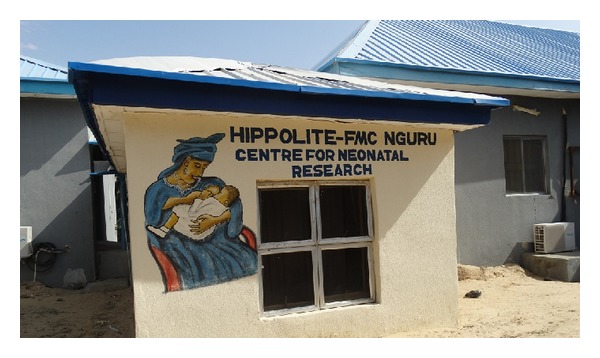
FMC Nguru research. This work investigated evening-fever syndrome in the neonate and recently published findings [9].

Academics within individual centres were invited to form local research groups and supported to carry out investigative studies of observable phenomena relating to temperature control in the neonate (Figure 4). A minimum of one possible research project was proposed to each participating centre accompanied with the provision of scholarly encouragements and affordable material support. This was a difficult concern as the culture of this kind of investigative research was unpopular amongst Nigerian clinicians and nurses. Only two individual centres were able to complete and publish any studies [9, 16]. Some of the successful efforts resulted in multicentre publications because the national outlook of this outreach made it possible to extract data on the same phenomenon across centres from different regions of Nigeria for comparison [11, 14]. Each centre was awarded 1 pt for every journal publication up to a maximum of 5 pts.

### 2.5. Concern-5: Localising Spare-Parts Fabrication

**Figure 5:**
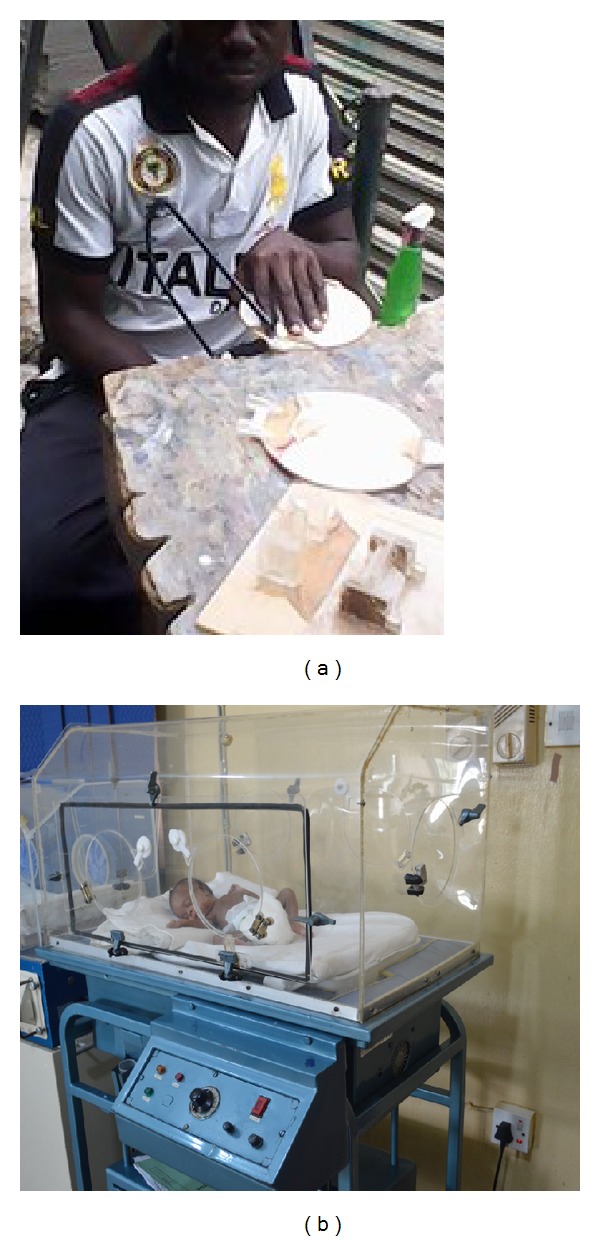
Localised spare parts fabrication. (a) An artisan working on Perspex items of incubator spare part in his private workshop. (b) Locally fabricated incubator in use at FMC Owerri.

Sustainability of the neonatal incubator practice amongst Nigerian SCBUs would be a difficult one if the question of maintenance of spare parts was not addressed. Local independent artisans were assessed and recruited for training wherever available within the cities where the participating hospitals were located. These included welders, painter, electricians, Perspex-craftsmen (Figure 5(a)), and tailors. These were trained on how to use their own skills and tools at the comfort of their own workshops to reproduce samples of various items of parts they were given. The produced items were assessed for quality control before usage. This approach made these parts affordable and readily available whilst creating jobs for the other members of the public. This method had initially been used to assemble a full locally made incubator that has been in use for over 5 years at the Federal Medical Centres (FMC) Owerri (Figure 5(b)). This was not assigned any centre scoring point because it was not based on how well a hospital performed.

### 2.6. Concern-6: Apnoea Monitoring and Nurses Availability

Neonatal apnoea episodes were highly frequent conditions in every Nigerian SCBUs corroborating with the country's well-known high neonatal mortality rate [4]. Apnoea might happen as a result of various physiological and clinically diagnosed or undiagnosed factors wilting the neonate. This might involve just one or repeated episodes before baby died from there. Early detection was crucial for baby's survival. Hence this must be monitored. There was no presence of apnoea monitors in any of the centres at the inception of the present outreach. Detection of the very frequent attacks was by “eye balling” by the highly overworked nurses. The neonates were so many but attending nurses so few per rota shift, so eye balling was inherently inefficient.

Nursing staff strength of the centres was so poor that none of the centres could demonstrate any better nurse-to-neonate ratio of 1 : 10. A typical Nigerian busy centre could witness persistent apnoeic attacks on up to 5 babies at the same time. This real life scenario suggested a possibility of losing up to 4 or all of the 5. This was because at the average of 10-neonate-workload, a nurse attending to the first apnoeic baby might not be aware of the other 4 early enough to save them. Therefore the hospitals were encouraged to (1) work towards 1 : 4 ratio as a minimum operational standard by employing more nurses and (2) install individualised cot/incubator fixable apnoea monitors. Market research for an appropriate design and affordable baby breathing/motion monitor was carried out. This yielded a recommendation to apply the BM02 apnoea monitoring system (Jablotron, Czech Republic) on all cots and incubators as minimum standard.

Nurse-to-neonate ratio of up to 1 : 4 was given the full 5 pts, up to 1 : 6 (4 pts), 1 : 7 (3 pts), 1 : 8 (2 pts), and 1 : 9 (1 pt). Apnoea monitors installation up to 3 Nos received 1 pt, up to 5 Nos (2 pts), up to 10 Nos (3 pts), up to 15 Nos (4 pts), and up to 20 Nos (5 pts).

### 2.7. Concern-7: Power-Banking

The introduction of RIT-incubator and its subsequent application in expanding the capacity of Nigerian SCBUs was intended to ensure availability of adequate number of functional incubators to save more babies. This aim soon began to be frustrated by impoverished mains power supply to operate the incubators when needed. Neonatal incubation being a “life support intervention” ought not to be switched off to preserve the neonate's life. Unannounced power cuts in Nigeria were very frequent and rampant and could last longer than 10 hours for each episode. Hence, neonatal hypothermia defined as body temperature below the physiological range of 36.5°C–37.4°C continued to be a serious concern even when baby managed to secure accommodation in available incubator. Hypothermia is associated with high neonatal mortality as has been well published [17]. A new standard that would localise power support was devised. The idle period in-between power availability was hence narrowed by the introduction of “power-banking” technique as a practice standard. This was achieved by the application of “3.5 KVA/48 Amps inverter-battery” system (Fussion series, Su-Kam industries India) for every group of up to 8 incubators and Resuscitaires. This was capable of sustaining these systems for up to 10 hours after power failure. This ensured uninterrupted neonatal incubation at the centres that adopted the idea. The presence of functioning power-bank in a centre earned 3 pts. If power-bank capacity was increased to power up to 16 or more incubators then the full 5 pts were awarded.

### 2.8. Concern-8: Failure-Preventive Audit Culture (FAC)

Incubators were operated nonstop, day-in day-out, so long as there was an occupant neonate. Hence, this needed to be regularly serviced to guarantee the prevention of avoidable breakdowns. Nigeria had a well-known culture of running systems and appliances to destruction due to neglecting professional maintenance. This often led to long periods of system out-of-use whilst management scrambled all over the place in search of repair or replacement. This cultural weakness affected the SCBUs too and resulted in unsustainable practice. FAC was introduced as a service of “professional advice when needed” accompanied with on-site “routine system assessment/maintenance.” This was a collaboration of professional care that managements were encouraged to enter into via the signing of Agreements or memorandum-of-understanding. This automatically empowered a highly reputable third party dedication that ensured the sustainability of the centre's incubator capacity. Routine maintenance was a mandatory 6-month exercise. Professional advice was executed whenever this was called for via electronic communication throughout the year. This ensured centre's consistency and sustainability of attained operational capacity. A score of 5 pts was assigned to centres with unbroken FAC service within the last 3 years leading up to March 2013.

### 2.9. Concern-9: Performance Competition

Annual performance competition was introduced in 2010 to award prizes to (1) the nation's overall best SCBU manager (normally the chief nursing officer of the winning SCBU) and (2) the hospital with the best managed collective group of SCBUs. Each centre was assessed twice a year at 6-month interval for an average annual score but without prior notification of when this would happen in order to capture the actual practice standards of the centre. The 1st and 2nd best hospitals and the best overall manager each received a trophy and various amounts in cash reward. The announcement of the “worst SCBU” of the year was introduced in 2013. This was to deter the nonchalant ward-managers and centres from complacency. The quest to be announced as “best SCBU” or “best manager” soon triggered competitiveness that seemed to have positively affected overall practice outcome across the participating centres. Heads of hospital managements were also taking pride in showcasing their strengths in different aspects of the concerns pursuit. The full 5 pts down to 1 pt were given to any centre that achieved 1st up to 5th position in each of the inclusive 3 years in this analysis (i.e., 2011–2013); average of scores for the three years was awarded.

### 2.10. Comparative Assessment

It was expected that positive impact of these concern remedies over time could alter prevailing status of indices such as neonatal mortality rate (NNMR), length of hospitalisation of surviving neonates, and patient influx [14]. The centres were assessed through retrospective data collected from their patient admission/discharge registers. By standards this was supposed to be a summary note-book that captured information such as dates of admission and discharge, birth-weight, gestation age, and overall outcome (dead or alive). This was supposed to be assiduously updated weekly with monthly summaries. Assessment did not require patient's identity to be revealed; however, all participating hospitals were invited to follow their institutional ethical rules to submit their data. Data submission was open to all 12 centres that currently held active “consultancy agreement” of the outreach. Three other big referral hospital centres that were not participating in the outreach were also invited to submit assessment data as “study controls.” The invited control centres represented the common practice status of a typical Nigerian SCBU and very similar to the states of the outreach centres before intervention began.

Submitted sets of data were used to measure three basic indices of clinical success, that is, overall average neonatal mortality rate (NNMR), average length of hospitalisation for the surviving neonates, and the associated patient-traffic (influx). The class of babies that might require incubator intervention for survival, incubator-dependent-neonates (IDN), was defined based on the Nigerian standards of neonatal classification. This included babies born earlier than 37 weeks of gestation (preterm) or those of birth-weight less than 2500 g (low birth weight). Intrauterine growth restricted (IUGR) babies within this boundary were also included provided birth-weight was less than 2500 g. Other quantified parameters were the fractional size of IDN babies in the presented population and the mortality rate of the IDNs (IDNMR). The total number of IDNs that died within 48 hours of presentation (d48 babies) was calculated and used to assess how overall NNMR was affected by these. Average duration of hospitalisation of the surviving IDNs was also quantified. One set of data was to be submitted by each participating centre to measure these performance indicators for the last 24 months leading up to March 2013, tagged post-RIT period. Outcomes were compared amongst outreach centres and also against outcomes from “control” centres.

The IDN class was further examined to identify the “very low” birth parameter cases; that is, very-LBW (≤1500 g) and very-preterm (≤32 weeks GA) cases. This was applied to investigate how very-low birth parameter cases might be currently affecting overall IDN survival statistics at the hospitals.

## 3. Results 

**Figure 6:**
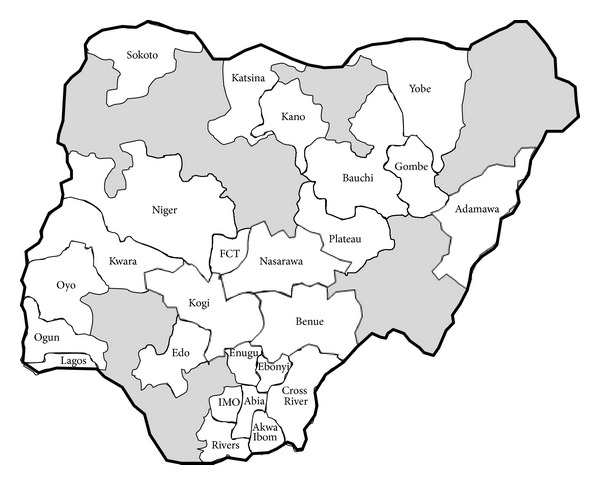
The states of Nigeria where any aspect of the present outreach has been carried out at their premier referral SCBU. Every Nigerian state has either one federal government university teaching hospital or one federal government medical centre that serves as the premier referral hospital for the state.

The managements of a very few tertiary hospitals in Nigeria accepted to try out the new ideas of recycled incubator technology (RIT) initially. These hospitals were however well-distributed across the regions of Nigeria. All the neonatal care centres responded positively with enthusiasm as the various procedures were being initiated in each centre. Most hospitals were able to recycle all their available dysfunctional/abandoned incubators. Others increased their capacity further by acquiring foreign used casings for recycling. The present coverage of the use of RIT systems or procedures across hospital centres in the states of Nigeria is shown in Figure 6. Many doctors and nurses enrolled for the training courses at various times; some repeated the courses and the associated examinations until they passed them.

**Figure 7:**
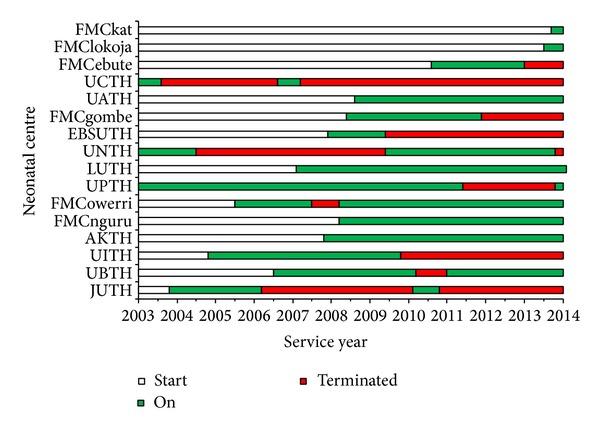
Failure-preventive audit culture (FAC) agreement timeline for the 16 tertiary hospitals that ever embarked on this up to the end of 2013. Neonatal centres were located at various Nigerian cities: FMCkat (city of Katsina), FMClokoja (Lokoja), FMCebute (EbuteMetta Lagos), UCTH (Calabar), UATH (Gwagwalada Abuja), FMCgombe (Gombe), EBSUTH (Abakaliki), UNTH (Enugu), LUTH (Idi-Araba Lagos), UPTH (Port Harcourt), FMCowerri (Owerri), FMCnguru (Nguru), AKTH (Kano), UITH (Ilorin), UBTH (Benin-city), and JUTH (Jos).

**Figure 8:**
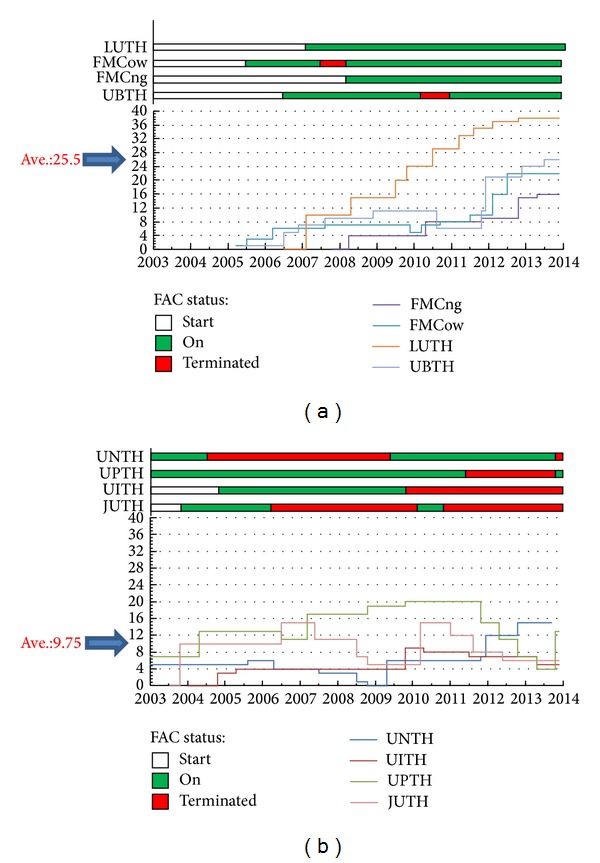
Incubator capacity growth or dwindle timeline. (a) Hospitals with little failure-preventive audit culture (FAC) disruption. (b) Hospitals with lengthy FAC disruption. *Y*-axis represented the total number of functional incubators. Neonatal centres were located at various Nigerian cities: FMCow (city of Owerri), LUTH (Idi-Araba Lagos), FMCng (Nguru), UBTH (Benin-city), UNTH (Enugu), UITH (Ilorin), UPTH (Port Harcourt), and JUTH (Jos).

A good number of the hospitals signed the agreements and operated the failure-preventive audit culture (FAC) component of the outreach (Figure 7). The managements of few hospitals ensured unbroken FAC services, some lasting up to 8 years to the time of this report. FAC was broken a number of times in other hospitals due to administrative successions that never considered this very important but restarted after it became obvious that stoppage had resulted in many SCBU disasters. We found that centres with less unbroken FAC operated with more consistent or increasing incubator capacities (Figures 7 and 8).

We observed that there was better structural transformation in hospitals where the management showed higher level of commitment to the entire outreach projects by strictly following their 6-month PAC reports and implementing them. Periodic interdepartmental reshovelling of nursing staff as practiced in Nigeria no longer affected SCBU trained nurses in hospitals where management mandated the proactive participation of the nursing department in the outreach project. There was better improvement of nurse-to-neonate ratio in these hospitals too. The annual national SCBU assessment competition motivated matrons and centres to achieve excellence. We observed that the best performing matrons/nurses were the most regular on courses and seminar attendance. The poorly performing centres in the national competition were mainly those that paid little attention to training courses irrespective of the growth of their incubator capacity. Only two individual centre groups were able to fully complete an investigative research and publish the original journal articles [9, 16].

**Figure 9:**
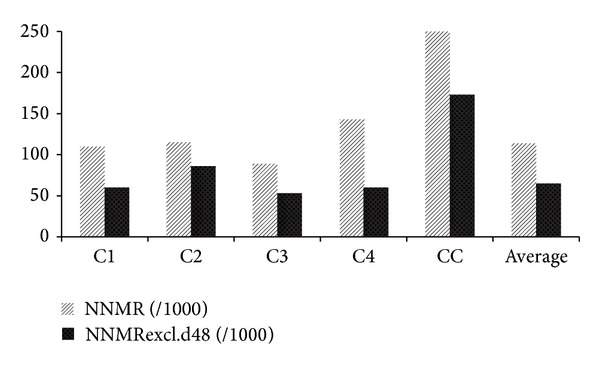
Mortality rate indices. NNMR: neonatal mortality rate; NNMRexcl.d48: neonatal mortality rate without neonates that die within 48 hours of presentation; average was based on the outreach centres.

Only 5 of 15 invited centres submitted their data for inclusion in the present analysis. These were from tertiary hospitals located in the southern Nigeria (C1 and C2), middle-belt (C3), and north (C4). We received submission from only one classified “control” centre (CC), a tertiary hospital in southern Nigeria (Figure 9). Average neonatal mortality (NNMR) across the outreach centres was computed at 114 deaths per 1000 neonatal admissions (114/1000). When d48 mortality was excluded, average NNMR reduced to 65/1000. Death rates were twice higher than these at the control centre (Figure 9).

**Figure 10:**
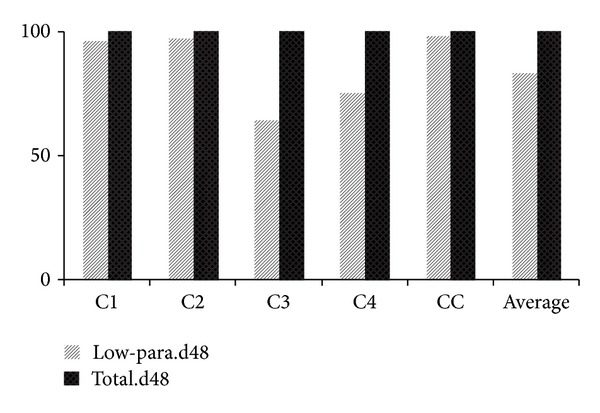
Neonatal mortality within 48 hours. “d48” stood for neonates that die within 48 hours of presentation, “low-para.d48” counted the size of very low parameter (≤1500 g or <33 wks) cases within the d48 baby population.

Typically, we computed that up to 64% of overall deaths of incubator-dependent-neonates (IDN) at the respective centres occurred within 48 hours of presentation. There were very few survivals amongst babies born before 30 weeks gestation or below a birth weight of 1000 g. Data also revealed that nearly all of the babies dying within the 48 hour window were either “born very early” or “born very small” (Figure 10). Average length of surviving IDN hospitalisation at the outreach centres was 20.5 days (average centre range: 8–32 days). This was not quantified at the “control” as supplied data lacked such details.

## 4. Discussion 

The recycled incubator technology (RIT) and associated outreach concerns in this study have demonstrated the significant capability of Nigerian special care baby units (SCBUs) in achieving improved newborn survival. An earlier publication to assess the impact of this Outreach in 2009 studied performances of the same group of hospitals that were involved in the present analyses [14]. This revealed that facility-based average NNMR had dropped from 254/1000 to 198/1000 babies [14]. This figure was only a reflection of what the country could have achieved if all the Nigerian hospital SCBUs adopted the same techniques as those analysed in the study. The NNMR of our “control” centre in the present study (250/1000) was very similar to the 254/1000 that was quantified as national average at the inception of the present outreach project as reported by Amadi et al. [14]. This is a validation that seemed to suggest that facility-based NNMR within a typical tertiary centre in Nigeria might remain within this high figure until the statuses of the concerns that have been identified in this study were positively altered.

**Figure 11:**
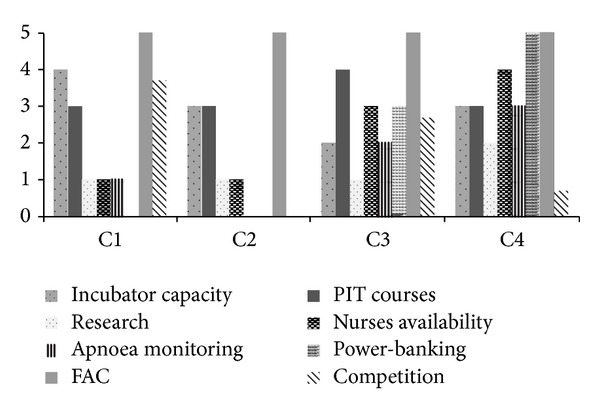
Concerns remedy implementation degrees. Scores: poor (0) to good (5).

At the inception of invitation to submit data for a retrospective assessment of the present outreach, all the hospitals invited to submit raw-data accepted with enthusiasm promising to keep up with the provided deadline. However, many of these either failed to comply or out rightly excused themselves from the study for various weak reasons, some of which might have stemmed from the fear of exposing suspected relative poor performance. The few submissions however had the advantage of coming from hospitals in the southern, northern, and middle-belt of Nigeria, giving a good national coverage. Our present study has shown that there has been further improvement on the overall outcomes of facility-based performance indices of the outreach centres. Notably, the average NNMR within the facilities has dropped from the figure of 198/1000 as reported by Amadi et al. [14] to 114/1000 in the present study. This significant leap must have been a result of the unprecedented dedication to capacity expansion, training, and implementation of FAC audit reports by the centres that have been assessed. Analysis was initially designed to use a scoring technique to comparatively measure the performances of entire centres in each of the concerns and to relate these to their respective outcomes. Unfortunately, the analysed data came from just a few of the participating hospitals for which very strong conclusions might not be drawn. However these were the top performing hospitals in the use of the concern remedies (Figure 11), and clinical and nursing practices in the centres had not been altered in any other direction since the 2009 assessments of Amadi et al. [14]. Hence result could only demonstrate that these concerns were the very vital components that brought about a significant reduction in NNMR as compared to the control.

**Figure 12:**
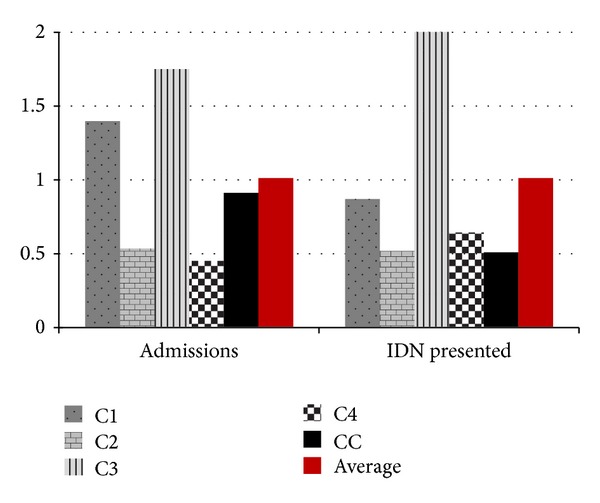
Patient influx. IDN stands for incubator-dependent-neonates.

**Figure 13:**
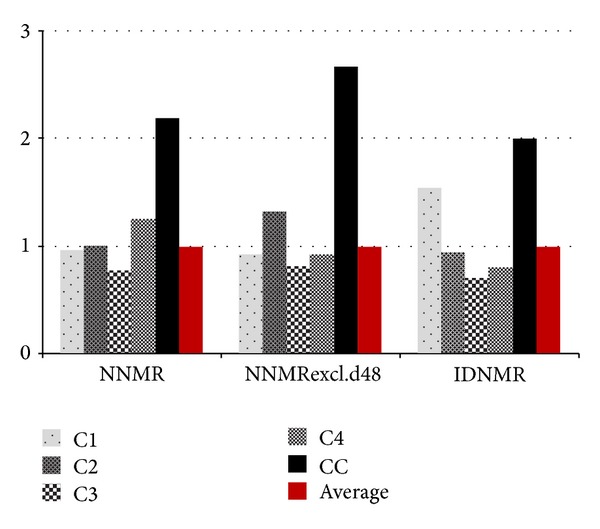
Performance indices comparison across all centres. NNMR stands for neonatal mortality rate, NNMRexcl.d48 for neonatal mortality rate excluding babies dying within 48 hours of presentation, and IDNMR for incubator-dependent-neonate mortality rate.

**Figure 14:**
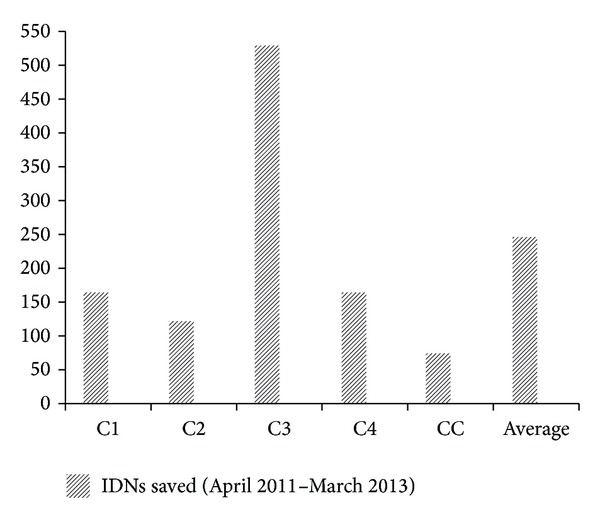
Total number of incubator-dependent-neonates that survived during the two-year period. “Average” was based on the outreach centres only.

We are not able to accurately quantify the relative contribution of each concern remedy towards the overall lowering of NNMR as these top centres applied varying levels of priority to different concerns. For example, the FMC Owerri was enabled to execute up to 7 concerns but particularly favoured continuous retraining of their SCBU staff while LUTH Lagos maximised 5 concerns and particularly favoured incubator capacity expansion up to 38 units of functional incubators and 8 units of Resuscitaires. This boosted LUTH's overall baby influx, a turnover of over 150 neonates in some months between patients being admitted in the “out-born,” “in-born.” and “paediatric-surgery” sections. FMC Nguru maximised all available remedies on the outreach without necessarily delimiting any. The admission registers of these high performing centres revealed higher influx of neonates as compared to situations prior to the present outreach. Overall baby admission at our control was comparable to other centres and had the third largest neonatal admissions (Figure 12). This suggested that the population within the catchment zone of this hospital had significant willingness to seek specialist intervention. However our control recorded the least relative number of IDN presentations amongst the admitted babies. This indicates a higher hesitation in the presentation of IDN babies to the control centre despite the people's good level of willingness to seek the hospital, perhaps as a result of very low success rate (Figure 13). The control centre (CC) saved the least number of IDNs (74 neonates) amongst the 5 individual centres over the 2-year period that was analysed. This was far smaller than the worst record from any of our outreach centres despite the control's locational and academic advantages as a teaching hospital (Figure 14). The clinical team at our control centre argued that the reasons for the poor outcomes “might be due to depletion of both infrastructure and skilled manpower as most of the incubators in our SCBU were broken down and the few functioning are in serious need of maintenance; also skilled nurses who have training in neonatal care were seldom given the opportunity to remain in the neonatal ward due to reshovelling of nurses around departments irrespective of the special training a nurse might have.”

Our outreach centre (C4) was probably the most remotely located federal referral hospital in Nigeria being situated at extreme northeastern region that was well known for higher infant mortality rates and people's unwillingness to seek hospital care [17]. This Centre was highly disadvantaged in terms of its very poor relative number of qualified doctors and nurses, without any fulltime consultant paediatrician. Illiteracy was relatively higher amongst mothers; hence these were very unlikely to seek early medical intervention, orchestrating the common situations of very poor neonatal vital-signs at the point-of-admission. Despite all these C4's index in terms of IDNMR was below average rate and was only outperformed by one Centre (Figure 13). It was our opinion that the overall reduction of average NNMR from 254/1000 to the present 114/1000 amongst our outreach centres was a demonstration of the effectiveness of the provided concern remedies.

The present study has also demonstrated the information regarding the neonatal deaths occurring within 48 hours (d48) of presentation. We would suggest that our analysed outreach centres had almost pushed the boundaries of neonatal mortality to the limits except for the d48 babies which constituted up to half of the total mortality in some centres. Most (83%) of the d48 babies were either very LBW or very preterm cases. A further investigation is hence recommended to urgently study the manner of care given to this class of patients, especially in the first 7 days of life, in order to synthesise any possible techniques that might guarantee better survival rate. The corporate Nigeria might not have achieved the MDG4 target; however our study has shown that there were pockets of individual hospital centres that have already surpassed this target. For example, our outreach centre C3 has achieved overall NNMR of 89/1000, a reduction of 65% in terms of the national facility-based average as computed in 2009 [14]. It would have been a possibility for Nigeria to corporately attain the MDG4 goal had the country paid more attention to the very simple but high impact approaches that were implemented at these few centres.
